# Updated Views in Targeted Therapy in the Patient with Non-Small Cell Lung Cancer

**DOI:** 10.3390/jpm13020167

**Published:** 2023-01-17

**Authors:** Miguel A. Ortega, Leonel Pekarek, Fátima Navarro, Oscar Fraile-Martínez, Cielo García-Montero, Miguel Ángel Álvarez-Mon, Raúl Diez-Pedrero, María del Carmen Boyano-Adánez, Luis G. Guijarro, Silvestra Barrena-Blázquez, Ana M. Gómez-Lahoz, Sergio Haro, Mónica Arroyo, Jorge Monserrat, Miguel A. Saez, Melchor Alvarez-Mon

**Affiliations:** 1 Department of Medicine and Medical Specialities, Faculty of Medicine and Health Sciences, University of Alcala, 28801 Alcalá de Henares, Spain; 2 Ramon and Cajal Institute of Sanitary Research (IRYCIS), 28034 Madrid, Spain; 3 Cancer Registry and Pathology Department, Prince of Asturias University Hospital, 28806 Alcalá de Henares, Spain; 4 Oncology Service, Guadalajara University Hospital, 19002 Guadalajara, Spain; 5 Oncology Service, Prince of Asturias University Hospital, 28806 Alcalá de Henares, Spain; 6 Department of General and Digestive Surgery, General and Digestive Surgery, Príncipe de Asturias Teaching Hospital, 28805 Alcalá de Henares, Spain; 7 Department of Surgery, Medical and Social Sciences, Faculty of Medicine and Health Sciences, University of Alcalá, 28801 Alcalá de Henares, Spain; 8 Unit of Biochemistry and Molecular Biology, Department of Systems Biology, University of Alcalá, 28871 Alcalá de Henares, Spain

**Keywords:** non-small cell lung carcinoma, lung adenocarcinoma, pulmonary squamous cell carcinoma, large cell carcinoma, immunotherapy

## Abstract

Non-small cell lung cancer (NSCLC) is the most frequent form of lung cancer and represents a set of histological entities that have an ominous long-term prognosis, for example, adenocarcinoma, squamous carcinoma and large cell carcinoma. Both small cell and non-small cell lung cancer are the main causes of oncological death and the oncological diseases with the highest incidence worldwide. With regard to clinical approaches for NSCLC, several advances have been achieved in diagnosis and treatment; the analysis of different molecular markers has led to the development of new targeted therapies that have improved the prognosis for selected patients. Despite this, most patients are diagnosed in an advanced stage, presenting a limited life expectancy with an ominous short-term prognosis. Numerous molecular alterations have been described in recent years, allowing for the development of therapies directed against specific therapeutic targets. The correct identification of the expression of different molecular markers has allowed for the individualization of treatment throughout the disease course, expanding the available therapeutic arsenal. The purpose of this article is to summarize the main characteristics of NSCLC and the advances that have occurred in the use of targeted therapies, thus explaining the limitations that have been observed in the management of this disease.

## 1. Introduction

Lung cancer is the second most common malignancy in the world, being the first most common in men and the second most common in women. It is estimated that, in 2020, there were more than 2.2 million new cases in the world, resulting in more than 1.8 million deaths globally; additionally, approximately 6% of the population will suffer from lung cancer throughout their lives [1]. Lung cancer is the first cause of death attributed to an oncological disease. The description of tobacco, radon, asbestos and other etiological agents as risk factors for NSCLC have led to the development of different public health campaigns that have been accompanied by a decrease in the incidence in recent years. Other risk factors for lung cancer have been described and include a multitude of agents, such as, arsenic, benzopyrenes among others; however, tobacco is responsible for up to 90% of lung cancer cases [2]. The release of polycyclic hydrocarbons, nitrosamines, nitrates and other carcinogens during combustion cause alterations in DNA repair mechanisms and cell cycle control and promote dysplasia processes that lead to malignant histological degeneration where the invasion and proliferation of malignant aberrant cells is predominant [3]. Notably, the most effective measure to reduce the mortality and incidence of lung cancer is the cessation of tobacco consumption. For example, at 10–15 years, there is a marked decrease in the relative risk of lung cancer. While the relative risk reduction for smokers is 81.4% after 1 year of smoking cessation, at 15 years, the relative risk reduction for lung cancer is 26%, indicating a clear benefit in the cessation of tobacco consumption [4]. Importantly, tobacco consumption is still frequent nowadays, given that 1100 million people smoke worldwide, and among them, between 10%–20% of smokers will develop lung cancer [5]. Lung cancer is more frequent among men but there has been evidence of an increase in the incidence of lung cancer in women in relation to different life changes in recent decades, and the maximum incidence by age occurs between 80–90 years, often accompanied by comorbidities and limiting the use of surgical options or aggressive chemotherapeutic regimens [6,7,8,9].

Lung cancer is differentiated into small cell lung cancer (SCLC) and non-small cell lung cancer (NSCLC), with the latter including lung adenocarcinoma, pulmonary squamous carcinoma, and large cell carcinoma. This classification is based on differences in the prognosis and clinical management between the two groups of entities [9].

On the one hand, SCLC represents approximately 20% of lung neoplasms. It is a very aggressive neoplasm characterized by rapid growth and presents extrathoracic metastases in more than half of patients at the time of diagnosis, making this entity, as a general rule, not susceptible to surgical oncological treatment [7]. This neoplasm is the most associated with paraneoplastic syndromes (Cushing syndrome, SIADH, gastrin secretion, VIP or Lambert-Eaton syndrome). Characteristically, they are cells of small size with scarce cytoplasm and salt-and-pepper chromatin centrally located [8]. It is usually treated with regimens that include etoposide and cisplatin and prophylactic cranial irradiation to reduce the development of brain metastases. Its treatment is therefore exclusively based on chemotherapy regimens, although in recent years, a variety of spindle and polygonal cells have been subjected to surgical excision, but its poor long-term prognosis is remarkable [9].

On the other hand, NSCLC represents the remaining 80% of all lung neoplasms. In recent decades, the discovery of different molecular targets has allowed for the development of targeted therapies against NSCLC, identifying groups of patients who are candidates for individualized treatments. Despite numerous advances, the average survival rate of patients with NSCLC is approximately 23%–27% at five years, exhibiting high lethality in addition to substantial comorbidity because most of these patients are diagnosed in advanced stages, when curative surgical options are limited [10]. Notably, the mortality associated with this entity is similar in both developed and developing countries, indicating that its prognosis is ominous despite the advances made and the availability of new treatment regimens. In recent years, numerous advances have been made in thoracic surgery for the management of these tumors in early stages using minimally aggressive techniques. Despite this, the five-year survival rate for patients with localized tumors is 64%, indicating the aggressiveness of these tumors despite being diagnosed in early stages. Given that NSCLC occurs in advanced ages, most patients present comorbidities that limit the surgical approach. In addition, more than 70% of patients who are diagnosed with NSCLC present locoregional or metastatic lymphatic dissemination, decreasing the probability of survival at five years to ~33% and ~7%, respectively [11,12]. Histological varieties of NSCLC have been traditionally classified by prognostic, anatomopathological and therapeutic factors. NSCLC groups together neoplasms being the most frequent squamous cell carcinoma, large cell carcinoma and adenocarcinoma of the lung. Herein, we will focus first on the different subtypes of NSCLC before deepening on the molecular alterations, clinical management and targeted therapies.

## 2. Subtypes of Non-Small Cell Lung Cancer

### 2.1. Squamous Cell Carcinoma

Squamous or epidermoid carcinoma is the subtype most associated with tobacco exposure, representing up to 30% of NSCLC [13]. This subtype is more frequent among males and is associated with the best prognosis, being centrally located in the upper lobes, tending to cavitate in up to 20% of cases and being associated with characteristic forms of paraneoplastic syndrome, such as Pancoast syndrome and the ectopic production of PTH [14]. At the anatomopathological level, it usually presents corneal pearls and keratin production, and both its staging and treatment are grouped in NSCLC.

### 2.2. Lung Adenocarcinoma

According to authors such as Provencio et al. [13], lung adenocarcinoma (LAC) currently represents more than 60% of non-small cell tumors. The incidence of LAC is similar among men and women, and its relationship with tobacco is not as strong as the relationship between tobacco and squamous cell carcinoma; it is the pulmonary neoplasia with the highest incidence in people who have never smoked and in young people under 45 years of age. In addition, it represents the most frequent cause of solitary pulmonary nodules and is associated with risk factors including pulmonary scars due to tuberculosis or asbestosis [15]. Histologically, it is composed of bronchial glands with a tendency toward a papillary configuration that degenerate by the proliferation of type II pneumocytes, generating atypical alveolar hyperplasia, and subsequently, invasive neoplasia. The WHO 2021 classification allows differentiating lesions according to their invasive potential, i.e., minimally invasive mucinous or nonmucinous lesions and nonmucosal invasive lesions, where four subtypes can be differentiated, such as acinar, papillary, lepidic, micropapillary and solid adenocarcinomas, invasive mixed mucinous lesions and others much less frequent such as colloid, fetal, enteric adenocarcinomas, each with diagnostic peculiarities, prognosis and management [16].

### 2.3. Large Cell Carcinoma

Anaplastic or large cell carcinoma usually represents between 5%–10% of NSCLC and usually presents peripherally associated with extrathoracic metastases in up to 80% of cases [17]. The WHO classification includes different subtypes, such as basaloid, large cell or neuroendocrine differentiation, among others. Its diagnosis is complex and requires the exclusion of different variants of epidermoid carcinoma or adenocarcinoma, for example, negativity for TTF1 or other immunohistochemical markers, which explains why, in the era of immunohistochemical diagnosis, 3% of NSCLC cases are actually large cell carcinoma [18].

The early diagnosis of this disease has been studied in high-risk patients (heavy smokers); there are screening programs that have been evaluated by low-dose computed tomography of the thorax and approved by the US Preventive Task Force but that in clinical practice are difficult to apply, causing the majority of patients to be diagnosed at advanced stages of the disease [19]. Regarding the management of pulmonary nodules, CT, thoracoscopy, mediastinoscopy and PET-CT have allowed for improvements in diagnosis in the initial stages for these patients; however, those who benefit still represent a small proportion and require complex management, exposing patients to a high level of emotional stress with follow-ups that can be prolonged for several months. The general treatment of NSCLC in initial stages I, II and IIIA, when the tumor is susceptible to surgical treatment, patients can undergo radiotherapy, neoadjuvant chemotherapy and subsequent surgery if they are surgical candidates. In more advanced stages, such as IIIB and IV, when there is mediastinal and subcarinal involvement, contralateral pulmonary invasion or metastatic dissemination, NSCLC is treated with chemoradiotherapy. Finally, we must highlight the development of immunotherapy because this approach not only represents a milestone in current oncology but has initiated a real revolution and expanded the therapeutic arsenal against cancer. Immunotherapeutic treatments are based on studies developed since the 1980s after the development of rituximab or CTLA 4 inhibitors [20]. In lung cancer, different assays have shown that the expression of immunohistochemical markers, genetic markers, molecular pathways and their relationship with the immune system are related to tumor proliferation. All this has improved the prognosis of selected patients with disseminated disease; however, more than 80% of patients diagnosed with advanced stage LAC do not survive more than five years [21]. Therefore, it is essential to study diagnostic and immunohistochemical markers that not only help with initial staging but can also be useful in the follow-up of patients in both advanced and early stages.

## 3. Molecular Markers in Lung Cancer

From the 2000s to present day, numerous clinical trials have shown the usefulness of different molecular markers. The initiation of immunotherapy in NSCLC is based on initial phase I trials (CheckMate 017 and 057 and Keynote 010) in which the response of immunotherapeutic immunomodulators against the program cell death receptor and its ligand in different neoplasms was evaluated, showing an increase in survival [22,23,24]. Given the good results observed, randomized clinical trials were conducted comparing chemotherapy with first-line immunomodulators for patients with NSCLC (such as Keynote 024 or CheckMate 026); based on the expression of PD-L1, the studies revealed the usefulness and possible limitations of immunomodulators, consolidating the evidence with regard to the use of immunotherapy in different neoplasms [25,26]. Moreover, numerous authors have described the existence of other molecular markers. For example, in 2004, Lynch et al. described mutations in the epidermal growth factor receptor (EGFR) that led to FDA approval in 2013 of erlotinib and gefitinib as first-line treatments for patients with NSCLC with advanced mutated EGFR [27]. The same occurred in 2007 after the discovery of anaplastic lymphoma kinase (ALK) rearrangements and the consequent approval of crizotinib (an ALK inhibitor) in 2011 by the FDA [28]. Since then, numerous molecular markers have been described to evaluate its efficacy, its diagnostic utility and its association with the prognosis of patients with NSCLC. All this has meant a change in the diagnosis and prognosis of patients with NSCLC. In 2015, the WHO introduced molecular markers in the diagnostic criteria of lung cancer. The update in 2021 of the WHO criteria has placed more emphasis on molecular markers [29]. According to general criteria, if a subgroup of patients are candidates for targeted therapies, all NSCLC samples are still studied regardless of the stage given the usefulness of the different drugs available. The treatment of disseminated disease has been based on the use of systemic chemotherapy regimens not only to limit the extension and limit tumor progression but also for palliative purposes to reduce the tumor burden and improve the symptomatology of patients in the final stages of the disease. Notably, the study of different molecular pathways has allowed an understanding of the underlying pathophysiology of the mechanisms of carcinogenesis, vascular invasion, proliferation and metastatic capacity. Like chemotherapy, immunotherapy is accompanied by numerous adverse effects that can be lethal and whose management is often complex.

The relevant molecular alterations with clinical implications are called driver mutations. Driver mutations are genetic alterations that occur in the preneoplastic phase of tumor cells and that confer a loss of cellular control and promote mechanisms of cell invasion and proliferation that allow for the development of invasive neoplasias. There are also passenger mutations, which have limited oncogenic effects and can be observed in nontumor cells [30]. Given the relevance of driver mutations, associations such as the WHO, College of American Pathologists, Spanish Society of Medical Oncology and Spanish Society of Pathology, among others, have recommended different diagnostic algorithms in relation to these biomarkers. In general, genotyping EGFR and BRAF V600E mutations, analyzing ALK and ROS1 rearrangements and investigating PD-L1 expression are recommended [31,32,33]. The mechanism for identifying different molecular markers is broad and includes techniques such as DNA sequencing, NGS (next generation sequencing), DNA allele-specific testing or simpler techniques such as immunohistochemistry or in situ fluorescence [34]. One of the most important milestones in the identification of driver mutations lies in the development of NGS. This has allowed for the study of a great variety of genes in different tumor lesions that can be analyzed to identify the clinical or prognostic relevance of different mutations, allowing for the generation of biomarker libraries that can be used in the future [35]. The identification of driver mutations in patients at different tumor stages depends on the availability of the previously described techniques, and in some cases, adequate time. For example, the average price per patient for NGS is 5000 dollars, and given that in Spain alone 24,000 cases of NSCLC are diagnosed each year, the cost would be approximately 120 million euros per year only for non-small cell lung cancer [36]. Other techniques have been studied, such as the identification of circulating tumor cells in peripheral blood in the case of genotyping cell-free plasma DNA or by plasma droplet digital PCR. Liquid biopsy is based on the concept of circulating tumor cells based on the existence of epithelial cells in the blood circulatory system derived after a process of angioinvasion and thus metastatic spread, which are not normally seen in patients without cancer. One CTC is usually found per 10 million leukocytes in peripheral blood. There are non-tumoral conditions in which CTCs are usually found in inflammatory diseases such as Crohn’s disease or endometriosis but to a lesser extent in tumor processes [37]. It should be noted that the importance of circulating tumor cells has already been described in prostate, breast and colon cancer, where their presence is associated with worse prognosis and higher recurrence rates after chemotherapy or surgery [38]. As we will see below, the identification of driver mutations using different techniques has meant a real advance in the treatment and prognosis of patients with non-small cell lung cancer. It is important to note that, despite the existence of targeted therapies, patients can progress due to immunoresistance to a given drug by acquiring new driver mutations, which would make it necessary to re-biopsy most patients. Conceptually, rebiopsy would be useful not only to study the mechanisms of immunoresistance (such as the T790M mutation in patients with EGFR-mutated lung adenocarcinoma) but also to understand the underlying pathophysiology of the molecular pathways that ultimately lead to immunoresistance [39]. The utility of liquid biopsy of peripheral blood would allow the detection of circulating tumor cells both to study new therapies and to create cell line cultures and better understand the physiological mechanisms of the metastatic process. It will also allow monitoring of immunochemotherapy treatment, as a decrease in circulating peripheral cells would indicate a response to treatment by the tumor [40,41]. In reference to lung cancer, where its importance has also been demonstrated both in the diagnosis and monitoring of these patients, authors such as Juan et al. or Pailler et al. have been able to detect ALK rearrangements in CTCs, which has allowed initiating therapy with tyrosine kinase inhibitors such as crizotinib, demonstrating its usefulness in the follow-up of patients diagnosed with lung cancer, although its sensitivity varies between 60–80%, which may limit its usefulness at the present time [42,43,44,45,46]. In recent years, advances in the genetic analysis of lung cancer have brought about a real revolution in the treatment and management of this disease. The possibility of obtaining the necessary material through liquid biopsy in peripheral blood to assess the immunohistochemical and genetic expression of peripheral tumor cells is accompanied by not only an improvement in the diagnosis of this disease, but also in the monitoring and early detection of immunoresistance mechanisms that cause the fatal outcome of these patients. In addition, on numerous occasions, patients have a large tumor burden, requiring the application of aggressive initial chemotherapy regimens to limit tumor growth. These techniques are not exempt from costs that sometimes cannot be assumed by health systems. Despite this, the therapeutic opportunity offered by the correct identification of driver mutations is one of the most important advances in current oncology and has allowed the initiation of a series of therapies that have led to a real change in precision medicine and have shown superiority over chemotherapy regimens used for years.

## 4. Targeted Therapy in NSCLC

Traditionally, the treatment of NSCLC has been based on platinum-based chemotherapy regimens, radiotherapy such as ablative stereotactic radiotherapy, and for indicated patients, surgical intervention [7,9]. During the last 20 years, numerous biomarkers have been developed, allowing the identification of therapeutic targets with the subsequent development of different targeted therapies. These therapies have shown efficacy and superiority over chemotherapy regimens in advanced stages through different clinical trials. Importantly, patients should be evaluated in a comprehensive way, and factors ranging from comorbidities to performance status should be taken into account to apply different lines of treatment. Regarding driver mutations, treatments can be differentiated based on the altered biomarker. Thus, targeted therapies are currently available for EGFR and BRAF mutations, ALK, RET and ROS1 rearrangements, MET alterations, and NTRK and anti-PD-L1 fusions, which will be explained below.

### 4.1. EGFR Mutations

EGFR mutations, initially described in 2004 by Lynch et al., cause the aberrant activation of a transmembrane tyrosine kinase and are present in 15% of NSCLC cases in our population (in Asian populations, the presence of EGFR mutations has been described in >50% of NSCLC cases), being more frequent in adenocarcinomas than in squamous cell carcinomas, in people with little or no smoking and in women [47,48]. EGFR mutations cause activation in different signaling pathways in metabolic pathways such as PI3K, JAK2, STAT3 and MAPK that cause increased cell proliferation and a loss of cell cycle control, resulting in uncontrolled growth [49]. EGFR gene alterations can be found in different exons, the most frequent being exons 19–21, each with different prognostic implications [50]. For example, mutations in exon 19 are more frequent (>61%) than those in exon 21 (~20%), with even higher values in different studies, as shown in a meta-analysis by Zhang et al. [51,52]. Currently, the first line of treatment for patients with advanced disease and EGFR mutations is based on first-, second- and third-generation tyrosine kinase inhibitors such as erlotinib, gefitinib, dacomitinib, afatinib and osimertinib, among others. First-generation drugs such as gefitinib or erlotinib were approved in 2015 by the FDA thanks to the NEJ 009, ENSURE, FIRST SIGNAL, OPTIMAL or LUX-lung 3 studies, which showed superiority in progression-free survival and median survival [53,54,55,56,57]. Subsequently, the ARCHER and LUX-Lung 3 studies investigated second-generation drugs, such as afatinib, approved in 2013 by the FDA, and dacomitinib [58,59]. Despite the use of tyrosine kinase inhibitors, the vast majority of patients present the T790M mutation, where a threonine is replaced by a methionine at position 790 of exon 20, resulting in an increase in ATP affinity for the EGF receptor, causing resistance to first- and second-generation tyrosine kinase inhibitors. In November 2015, and given the results of the AURA 3 study, the use of third-generation tyrosine kinase inhibitors such as osimertinib in patients with the T790 M mutations was approved [60]. Importantly, patients with brain metastases and EGFR-mutated NSCLC show a good response to third generation TKIs, which adequately control central nervous system infiltration [61]. Metastatic involvement of the CNS occurs in up to 25% of patients diagnosed, hence its clinical relevance. Currently, osimertinib is one of the most important tyrosine kinase inhibitors, given that clinical trials such as FLAURA, which included 556 patients with EGFR mutation, have demonstrated a median survival and a longer median duration of response compared to other tyrosine kinase inhibitors used in EGFR mutation [62]. However, the usefulness of osimertinib has been studied in those patients with T790M mutation pre-treated with other TKIs observing an objective response rate ranging from 49% to 66% with a progression-free survival of approximately 10 months according to different authors [63,64]. Actual recommendations indicate that the T790M mutation can be evaluated by liquid biopsy, or in cases of progression to treatment with tyrosine kinase inhibitors, the lung lesion can be rebiopsied to evaluate the presence of this mutation and therefore resistance to TKI [65]. If patients progress despite all lines of TKI treatment, the use of platinum-based chemotherapy should be considered [66,67]. EGFR alterations have led to the initiation of targeted therapy in lung cancer in a more standardized manner, and this alteration is the most frequent driver mutation. In addition, numerous mutations in different exons are being described with different prognostic values thanks to techniques such as NGS, demonstrating the clinical importance of the different molecular biology techniques available.

### 4.2. ALK Rearrangement

The chromosomal rearrangement of anaplastic lymphoma kinase (ALK) was first described in 2007 in anaplastic large cell lymphomas, and Soda et al. reported that it occurs in ~7% of patients with NSCLC [68]. It is much more frequent in adenocarcinomas than in squamous or large cell tumors, where its presence is very rare [69]. ALK rearrangement involves alterations in the signaling of a type of insulin-related receptor tyrosine kinase present in neurons of the central nervous system and has been described in numerous tumors, such as anaplastic long cell lymphoma, inflammatory myofibroblastic tumors and neuroblastoma. The rearrangement of ALK causes pathological activation of the EML4 (echinoderm microtubule-associated-protein-like 4)-ALK complex, leading to alterations in the correct formation of microtubules and the proliferation and migration of tumor cells [70]. Notably, ALK rearrangement presents special clinical characteristics. For example, patients with ALK rearrangement are more likely to develop brain metastases [71]. In addition, patients with ALK rearrangement are usually younger than patients without ALK rearrangement, with a lower mean age, and ALK rearrangement is more frequent in nonsmokers and patients who do not usually present other alterations in driver mutations [72]. ALK rearrangement responds to crizotinib, ceritinib or lorlatinib. For example, in a study that included 143 patients, Camdige et al. reported that a first-generation TKI (crizotinib) had a response rate of 60% with a progression-free survival of 9.7 months and a median response of 49 weeks, as compared with a response rate of 10% and a survival of three months for patients who received chemotherapy [73]. As with anti-EGFR drugs, there are resistance mechanisms that cause a decrease in efficacy at approximately one year, with the most frequent mutation being L1196M, an analog to EGFR T790M, in addition to others such as G1202R or S1206Y, which are amplifications of C-Kit, causing a decrease in the affinity of crizotinib for tyrosine kinase [74]. New generation tyrosine kinase inhibitors such as ceritinib or specially lorlatinib have shown superiority over crizotinib and are associated with greater progression-free survival and a better response in cases of brain metastasis (NCT03052608). In this line, we should highlight the CROWN clinical trial conducted by Shaw et al., which shows the efficacy of lorlatinib in patients with ALK rearrangement with response rates of 76% and response rates of 82% for metastatic brain disease, which demonstrates relevant efficacy in central nervous system involvement. [75,76]. However, one of the tyrosine kinase inhibitors currently used in patients with ALK mutations is alectinib. In this line, meta-analysis, such as that carried out by Tang et al., which includes three studies with up to 697 patients comparing alectinib with crizotinib, shows a clear superiority in terms of overall response rate and progression-free survival of alectinib [77,78]. In addition, authors such as Zou et al. have shown that alectinib is effective in controlling metastatic involvement in the CNS in ALK-positive patients, both symptomatic and asymptomatic, which implies an improvement in the prognosis of patients with brain extension [79]. Among the new therapies in cases of ALK translocation, we should mention the availability of brigatinib which, according to the results of the ALTA-1L clinical trial, not only shows superiority over crizotinib in terms of survival but also has a lower side-effect profile, which implies better tolerability [80]. Therefore, although ALK is a less frequent mutation, there are different lines of treatment that have shown great clinical utility in patients with ALK rearrangement.

### 4.3. ROS1 Rearrangement

In 2007, alterations in the oncogene c-ROS1 were described as driver mutations, leading to advances in the development of targeted therapies; these alterations are present in up to 2% of LAC [81]. This rearrangement, like the others, is much more frequent in patients with adenocarcinomas than with other histological varieties and is more common in young patients and nonsmokers. This mutation usually occurs between the ROS1 and CD74 oncogenes and is accompanied by the activation of metabolic pathways such as JAK/STAT, PI3K/AKT and MAPK/ERK, causing an increase in cell proliferation, cell survival and histological invasion capacity [82]. Currently, one of the therapies that has shown greater efficacy in these patients is the ROS1/MET tyrosine kinase inhibitor crizotinib. The results of the EUCROSS clinical trial (NCT02183870), which reported a mean survival at 48 months of 55% in 30 patients with NSCLC with ROS rearrangement, are consistent with those for the PROFILE 1001 clinical trial, in which 53 patients with NSCLC and ROS rearrangement were evaluated, with a response rate of 72%, a mean duration of response of 24.7 months and a median survival of 51.4 months [83,84]. The efficacy of entrectinib, an ROS1/tropomyosin tyrosine kinase inhibitor, has also been shown by different authors. For example, Dziadziuszko et al., in a clinical trial with 161 patients with NSCLC with ROS rearrangement and on therapy with entrectinib, reported a response rate of 67.1% with an intracranial metastasis response rate of 72.9% and a progression-free survival of 15.7 months [85]. As with TKIs with EGFR, there are immunoresistance mechanisms against crizotinib and entrectinib that have led to the evaluation of new TKIs. For example, the clinical trial NCT01970865 evaluated the efficacy of lorlatinib (third-generation TKI) in 69 patients with ROS rearrangement. The response rate for the 69% of patients who had previously received crizotinib was 35%, and the response rate for the 31% who had not previously received crizotinib was 62% [86]. While a small number of patients with ROS1 rearrangement are candidates for immunotherapy, the response rates are encouraging.

### 4.4. MET Mutations

Another of the relevant biomarkers in NSCLC occurs in the receptor tyrosine kinase associated with MET and its ligand hepatocyte growth factor, leading to the activation of pathways such as AKT, ERK/MAPK or STAT 3. The consequence is an increase in cell survival, proliferation and cell migration [87]. Metabolic pathway signaling in the MET-HGF cascade can be based on the overexpression of MET or HGF, the amplification of the MET gene or mutations and rearrangements in MET. Aberrant signaling in MET are usually more common in adenocarcinomas, highlighting exon 14 skipping mutations, present in up to 3% of non-small cell adenocarcinomas and a characteristic in up to 20% of sarcomatoid histology. MET gene amplifications can be observed in up to 4% of patients with adenocarcinomas and in up to 20% of those patients with EGFR mutations after immunosuppressive treatment [88,89]. The molecular mechanisms of the malfunction of the tyrosine kinase associated with MET with its ligand hepatocyte growth factor are accompanied by the activation of pathways such as AKT, ERK/MAPK or STAT 3, resulting in an increase in cell survival, cell proliferation and cell migration. The MET exon 14 mutation causes a decrease in MET degradation, generating its activation [90]. Currently, there are tyrosine kinase inhibitors that act on MET, for example, capmatinib, as shown by the GEOMETRY mono-1 clinical trial, with response rates of 68% in targeted therapy-naive patients and a median progression-free survival of 12.4 months after first-line treatment for NSCLC with MET mutation [91]. Other treatments, such as tepotinib, have shown, through the VISION clinical trial, response rates of approximately 50% in 152 patients, with a median response of 11 months. Crizotinib, a multiple tyrosine kinase inhibitor, acts on MET, ALK and ROS [92]. Drilon et al., in a trial with 69 patients with MET exon 14 skipping, reported a response rate of 32%, with a mean response duration of 9.2 months and a disease-free progression of 7.3 months [93]. However, MET amplifications may be due to de novo amplification or a consequence of immunoresistance mechanisms to therapies directed not only at TKI against MET but also against EGFR, with immunofluorescence in situ hybridization being the preferred technique to detect such amplifications [94]. Several authors have studied the use of capmatinib in patients with high amplifications derived from mechanisms of immunoresistance or chemoresistance. For example, the GEOMETRY mono-1 study showed that in patients with <10-fold amplification, responses were obtained in up to 12% of patients and that in patients with copies >10, responses were observed in 29% of patients previously treated with immunotherapy and in up to 40% of treatment-naïve patients [95]. Therefore, patients with MET alterations do not present responses to treatment that are as optimal as those for EGFR mutations, but they may benefit from targeted therapy.

### 4.5. RET Rearrangement

The rearrangement of the RET gene conditions the activation of cytosolic kinases derived from RET; this rearrangement occurs in 1%–2% of patients with adenocarcinomas [96]. Patients with RET rearrangement tend to be younger, with little or no history of smoking, and like EFGFR or ALK mutations, RET rearrangement is associated with a high probability of metastatic progression in the CNS [97]. RET rearrangement is susceptible to treatment with different TKIs. For example, the LIBRETTO-001 clinical trial evaluated the use of selpercatinib in different clinical situations, yielding promising results. In 105 patients with RET fusion who had previously received platinum-based chemotherapy, the response rate was 64%, with a median response of 17.5 months. In 39 treatment-naive patients with alterations in RET, the response rate was 85%, and for 90% of those patients, the response was maintained at 6 months. In 11 patients with CNS metastases, the intracranial response rate was 91% [98]. The ARROW trial evaluated the use of pralsetinib in 223 patients with alterations in RET. Ninety-two had previously received chemotherapy; their response rate was 61%, with 6% having complete responses. For patients who had not received chemotherapy, the response rate was 70%, with 11% having complete responses [99]. Other drugs, such as cabozantinib, vandetanib, alectinib and sunitinib, which are multiple kinase inhibitors, have lower efficacy in NSCLC and greater activity in thyroid neoplasms, where RET alterations are usually more frequent [100]. RET rearrangement has unique clinical characteristics (young people and light smokers with a high incidence of disease in the CNS), and immunotherapy has resulted in sufficiently acceptable response rates in these patients.

### 4.6. BRAF Mutations

Mutations have been described in the V-Raf murine sarcoma viral oncogene homolog B (BRAF) kinase; these mutations cause the activation of the MAPK pathway and are usually present in up to 4% of patients with NSCLC, being much more frequent than other driver mutations in adenocarcinomas [101]. The evolution of anti-BRAF therapies has been based mainly on patients with metastatic melanoma; these treatments have substantially improved the prognosis of these patients; therefore, their applicability in lung cancer has been studied. Two groups can be differentiated: Patients with V600 mutations or non-V600 mutations. Litvak et al. reported that among 63 patients with BRAF mutations, 57% had the V600E mutation, which was more frequent in light smokers or nonsmokers and women, and 43% did not have the V600E mutation, with a better prognosis for those with V600E mutation [102]. As in melanoma, the combination of dabrafenib and trametinib (BRAF and MEK inhibitors) acts in a synergistic manner against BRAF mutations and the underlying metabolic pathways. A clinical trial (NCT01336634) showed the benefits of the use of this combination in 93 patients (previously treated with chemotherapy or naive patients). For previously treated patients, the response rate was 68.4%, and for treatment-naïve patients, the response rate was 63.9%, with mean survival rates of 18.2 and 17.3 months, respectively [103]. This result indicates good clinical responses in previously treated patients and treatment-naive patients, with minimal differences between them. Patients who do not present the V600E mutation are not usually candidates for this combination; however, different authors have recommend administering it in the absence of sufficiently powerful clinical trials [104]. The use of different therapies overlaps in different neoplasms for the same marker, providing a similar clinical benefit. In this case, anti-BRAF melanoma immunotherapy has shown its usefulness in a small but no less important percentage of patients with NSCLC.

### 4.7. NTRK Fusions

Molecular aberrant expression in tropomyosin receptor kinases (NTRKs) are one of the least frequent driver mutations and occur in less than 1% of adenocarcinomas. NTRK fusion seems to act as a resistance mechanism against EGFR TKIs in patients with NSCLC [105]. Different authors have shown the efficacy of larotrectinib in patients with NSCLC and NTRK fusions [106]. For example, Drilon et al., who conducted a study that included 20 patients, reported a response rate of 20%, with a median duration of response of 35.4 months and a mean survival of 40.7 months, showing the benefit of this therapy, with good response and maintenance rates in the long term [107]. In addition, this therapy has shown efficacy in other types of tumors, such as thyroid cancer, sarcomas, colorectal cancer, central nervous system tumors, etc. Similarly, Doebele et al. evaluated the use of entrectinib in patients with NSCLC; the response rate was 57%, with a high average survival duration [108]. Despite being a very rare alteration, patients could benefit from a first-line drug that treats this driver mutation.

### 4.8. KRAS Mutations

Alterations in KRAS (Kirsten rat sarcoma viral oncogene homolog), NRAS (neuroblastoma rat sarcoma viral oncogene homolog) and HRAS (Harvey rat sarcoma viral oncogene homolog), the most frequent being the KRAS mutation, which has been described in up to 30% of patients with non-small cell lung cancer predominantly in the adenocarcinoma variant and in smokers [109]. The KRAS G12C mutation, which produces a variation of the glycine nucleotide for cysteine at codon 12, represents the most frequent form of mutation of the RAS gene, which generates an activation of the GTP-KRS compound, which leads to inhibition in signaling pathways of cell control and an increase in cell survival and tumor growth [110]. All this has led to different clinical trials to evaluate anti-KRAS therapies, which has led to the approval of irreversible KRAS G12C inhibitor drugs such as sotorasib or adagrasib. For example, the CodeBreak100 clinical trial that included 126 patients with metastatic non-small cell lung cancer, of whom 81% had received other targeted therapy with PD-1 or PD-L1 inhibitors, a response was observed in up to 37.1% of patients with a median response of 11.1 months and a median survival of 12.5 months with sotorasib [111]. However, the KRYSTAL-1 clinical trial has evaluated the usefulness of adagrasib in 116 patients with metastatic non-small cell lung cancer in 116 patients with the KRAS G12C mutation, practically all of whom had received chemotherapy and immunotherapy. In these patients, a clinical response is observed in up to 43% of patients, a duration of response of 8.5 months and a median survival of 12.6 months [112]. Diverse mechanisms of inmunoresistance in patient remains have been evidenced, being the most frequent mutations in secondary KRAS such as Y96C, H95Q, H95R, among others, being the main ones observed [113]. Given the high frequency of the KRAS mutation and the possibility of having targeted therapies recently approved by the FDA, the use of adagrasib and sotorasib are initial alternatives in patients with non-small cell lung cancer, also showing their usefulness in patients who have progressed to other lines of chemotherapy or immunotherapy.

## 5. Conclusions

**Figure 1:**
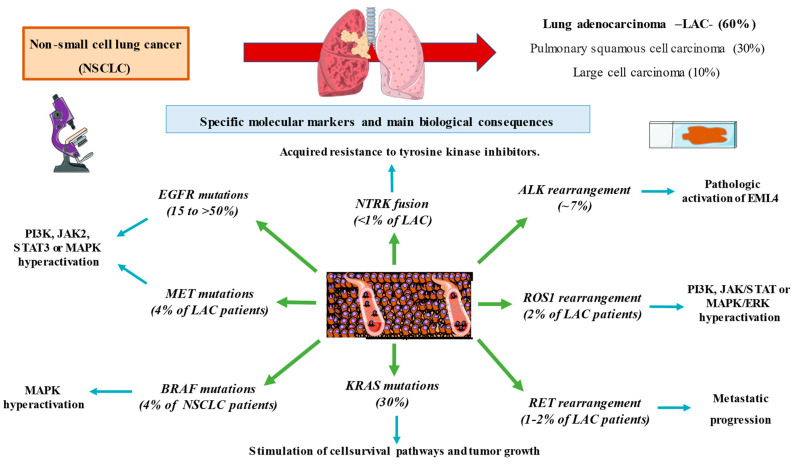
A global summary of the specific markers studied in non-small cell lung cancer and their derived biological implications.

**Figure 2:**
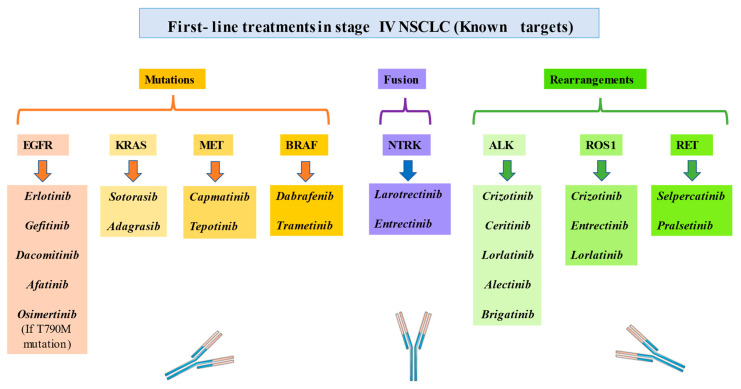
First-line treatments in patients with stage IV NSCLC with known target mutations, fusion and rearrangements.

Lung cancer is one of the most common and the deadliest neoplasm. NSCLC onset is associated with the consumption of tobacco as well as other risk factors, such as exposure to asbestos. Despite numerous efforts to study screening programs, most patients are diagnosed in advanced stages of the disease. In recent years, various molecular markers have been described determining certain biological features and explaining the behavior and progression of these types of tumors (Figure 1). In parallel, this has allowed the development of numerous targeted therapies (Figure 2) and resulted in positive changes in the prognosis of patients. The correct identification of these molecular alterations has revolutionized the management of this entity, radically changed the perception of immunotherapy, facilitating the development of individualized precision medicine where each patient is analyzed in a systematic but individual way (Table 1). With this, the future objective of the management of non-small cell lung cancer in its different histological expressions should be based on improving early detection, identifying new molecular pathways that are susceptible to targeted therapies and systematizing the different therapeutic options available. This approach will facilitate important advances in oncology.

## Figures and Tables

**Table 1:** Targeted therapies according to the molecular target and their effectiveness rate. (ORR: Overall response rate) (PFS: Progression-free survival in months). (OS: Overall survival in months).

Therapeutic Target (Percentage in NSCLC)	Targeted Therapy	Effectiveness	Reference
EGFR mutation (15–50%)	Erlotinib	ORR 83% PFS 13.1 months	[54]
	Gefitinib	ORR 84.6% PFS 8.4 months OS 30.6 months	[55]
	Dacomitinib	ORR 75.6% PFS 18.2 months. OS 34.1 months	[56]
	Afatinib	ORR 67.2% PFS 11.8 months	[57]
	Osimertinib	ORR 80% PFS 18.9 months.	[63,64]
ALK rearrangement (7%)	Crizotinib	ORR 60% PFS 9.7 months.	[73]
	Ceritinib	ORR 58% PFS 7 months.	[76]
	lorlatinib	ORR 76% PFS 18 months.	[75]
	Alectinib	ORR 90% PFS 34.8 months.	[77,78,79]
	Brigatinib	ORR 74% PFS 29.4 months	[80]
ROS1 rearrangement (2%)	Crizotinib	ORR 70% PFS 20 months	[83,84]
	Lorlatinib	ORR 62% PFS 21 months.	[86]
	Etrectinib	ORR 67% PFS 15.7 months.	[85]
MET mutation (2%)	Capmatinib	ORR 68% PFS 12,4 months	[91]
	Tepotinib	ORR 50% PFS 11 months	[92]
RET rearrangement (2%)	Selpercatinib	ORR 64% PFS 16.5 months	[98]
	Pralsetinib	ORR 61% PFS 7.3 months.	[99]
BRAF mutation (4%)	Dabrafenib and trametinib	ORR 68.4% PFS 10 months	[103]
NTRK fussion (<1%)	Larotrectinib	ORR 73% PFS 35.4 months.	[107]
	Entrectinib	ORR 57% PFS 11 months	[108]
KRAS mutation (30%)	Sotorasib	ORR 37.1% PFS 6.8 months	[111]
	Adagrasib	ORR 42.9% PFS 6.5 months	[112]

## Data Availability

Not applicable.
